# Distal cortical notching significantly decreases tibial stability following tibial tubercle osteotomy: An in vitro porcine biomechanical study

**DOI:** 10.1002/jeo2.70858

**Published:** 2026-07-17

**Authors:** Eiken Senkbeil, Kay Sellenschloh, Karl‐Heinz Frosch, Matthias Krause, Sara Checa, Jannik Frings

**Affiliations:** ^1^ Department of Trauma and Orthopaedic Surgery University Medical Center Hamburg‐Eppendorf Hamburg Germany; ^2^ Department of Trauma and Orthopaedic Surgery Military Hospital Hamburg Germany; ^3^ Institute of Biomechanics Hamburg University of Technology Hamburg Germany; ^4^ Department of Trauma Surgery, Orthopaedics and Sports Traumatology BG Hospital Hamburg Hamburg Germany; ^5^ Department of Orthopaedic and Trauma Surgery University Medical Center Schleswig‐Holstein, Campus Luebeck Luebeck Germany

**Keywords:** complication, fracture, instability, osteotomy, patella, patella alta, tibial tubercle transfer

## Abstract

**Purpose:**

Distalizing tibial tubercle osteotomy (dTTO) is an effective treatment for patella alta, yet complications can be severe. This study evaluates the biomechanical effects of distal cut orientation on tibial fracture risk. It was hypothesised that orthogonal cutting and cortical notching reduce tibial stability.

**Methods:**

A total of 61 porcine tibiae were tested in three setups. Four distal cut orientations were compared in a load‐to‐failure analysis (*n* = 32): oblique (Obl), orthogonal without notch (Orth), orthogonal 2‐mm notch (Orth2), orthogonal 4‐mm notch (Orth4) (each *n* = 8). Cyclic loading was evaluated in Obl, Orth and Orth2 (*n* = 17), followed by comparison of plate versus (2‐hole one‐third tubular plate) 3.5‐mm screw fixation in Orth2 specimens (*n* = 12). Specimens were embedded in PMMA and axially loaded (MTS Bionix 358). Load‐to‐failure, stiffness and cycles until failure were recorded. Residual tibial thickness was measured. Linear regression analysed the relationship between residual thickness and failure load. Statistical analysis included *t*‐tests and ANOVA (*α* = 0.05).

**Results:**

Obl showed the highest failure load (784.5 N ± 118.3), although the difference compared to Orth (538.8 N ± 229.5, *p* = 0.087) did not reach statistical significance. Notching significantly reduced load‐to‐failure (2‐mm: 294.0 N ± 91.8; 4 mm: 274.6 N ± 96.5). In cyclic testing, Obl withstood most cycles until failure (5894 ± 2187) compared to Orth (3142 ± 808; *p* = 0.004), while Orth2 failed sooner (1400 ± 530, all *p* < 0.005). Osteosynthesis techniques did not affect failure load (*p* = 0.802). Stiffness and residual tibial thickness were comparable. Distal tibial thickness was associated with failure load in all groups (all *p* < 0.05).

**Conclusion:**

In this porcine model, cortical notching significantly reduced tibial stability and should be avoided during TTO. Oblique distal cuts demonstrated greater resistance to cyclic loading, while no significant difference was observed in static load‐to‐failure. Osteosynthesis technique did not affect load‐to‐failure.

**Level of Evidence:**

NA, cadaveric biomechanical study.

**Abbreviations**
ANOVA: analysis of variance
dTTO: distalization of the tibial tubercle
PA: patella alta
PFI: patellofemoral instability
PMMA: polymethyl methacrylate
TKA: total knee arthroplasties
TTO: tibial tubercle osteotomy

## INTRODUCTION

**Figure 1:**
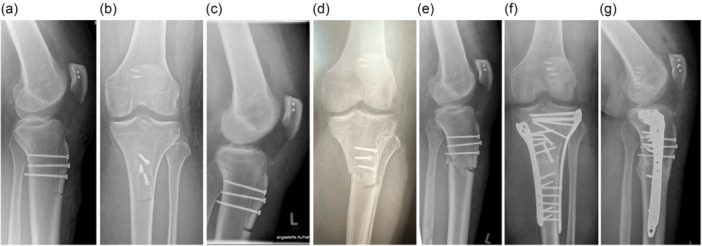
Left knee of a 27‐year‐old patient with patellar instability and maltracking due to patella alta. Tibial tubercle distalization and medialization were performed using an orthogonal distal cut and fixed with three bicortical lag screws (a). Six weeks postoperatively, no relevant changes occurred (b, c). Nine weeks postoperatively, the patient presented with acute pain after an inadvertent lunge on the operated leg, presenting a tibial shaft fracture at the level of the distal osteotomy cut (d, e). Six months after revision surgery, the tibial fracture had healed completely (f, g).

Patella alta (PA) is considered one of the most relevant risk factors for patellofemoral instability (PFI) [2, 4, 7, 11, 37]. Delayed engagement of the patella into the trochlear groove during early knee flexion reduces osseous stabilisation and increases patellofemoral contact pressures [8, 15, 23, 34]. In skeletally mature patients with symptomatic PA, distalization of the tibial tubercle (dTTO) is an established surgical treatment to improve patellofemoral biomechanics [9, 20, 24, 33]. Clinical studies have shown good to excellent outcomes following dTTO [18, 20, 31]. However, the procedure is associated with an overall major complication rate of up to 21.5% [13, 22, 29, 36]. A rare but serious complication is the tibial shaft fracture. Reported incidences vary in the literature, ranging from 0.4% to 3.7% of all cases [12, 17, 22]. Clinical reports have suggested an association between orthogonal distal osteotomy cuts and postoperative tibial fractures [1, 10, 31, 33] (Figure 1). In dTTO, an orthogonal distal cut orientation can help to achieve a standardised distal resection [19, 21]. At the same time, an orthogonal cut may act as a local stress riser at the anterior tibial cortex, especially with increased saw depth or unintended cortical notching [19, 28]. To avoid this, alternative osteotomy designs using an oblique distal cut have been proposed to reduce fracture risk [19, 21, 33]. However, biomechanical evidence supporting this idea remains limited. Previous studies have mainly focused on stability of tibial tubercle fixation techniques, but the effect of distal cut orientation on the perioperative fracture risk has not yet been explored. Furthermore, the potential of different osteosynthesis techniques to compensate for a subsequent reduction of bone stability remains unclear.

The purpose of this study was therefore to evaluate the effect of distal osteotomy cut orientation on tibial stability following dTTO, comparing orthogonal and oblique distal cut orientations in terms of load‐to‐failure and fracture behaviour. Furthermore, the biomechanical relevance of cortical notching and the stabilising effect of different osteosynthesis techniques under static loading conditions were investigated. The present study hypothesised that orthogonal cut orientation would result in reduced biomechanical stability and lower failure loads compared to an oblique cut, particularly when combined with cortical notching. It was further hypothesised that combined anterior plate osteosynthesis would provide greater stability compared to isolated screw fixation.

## MATERIALS AND METHODS

### Specimens

This in vitro study comprised three different experimental series, each of which evaluated differences between four distal osteotomy cut orientations and two fixation techniques. A total of 63 fresh‐frozen porcine tibiae were included in this study. One tibia served as a reference specimen in the load‐to‐failure analysis. Specimens were obtained from 7‐ to 8‐month‐old pigs from regional abattoirs in northern Germany. Porcine tibiae were selected because their cortical structure and mechanical properties are comparable to those of human tibiae and they are well established for orthopaedic biomechanical testing [25, 35]. The tibiae were vacuum‐sealed and stored at −19°C for a maximum of 8 weeks. Before testing, specimens were thawed at room temperature to allow complete removal of soft tissues. Each tibia was inspected for pre‐existing damage or macroscopic abnormalities and excluded if defects were identified. During preparation, specimens were kept moist using Ringer's solution, sealed in plastic bags and stored in a cooled container until testing. Throughout all preparation steps, specimens were continuously hydrated to avoid drying‐related alterations in material behaviour.

### Osteotomy techniques

**Figure 2:**
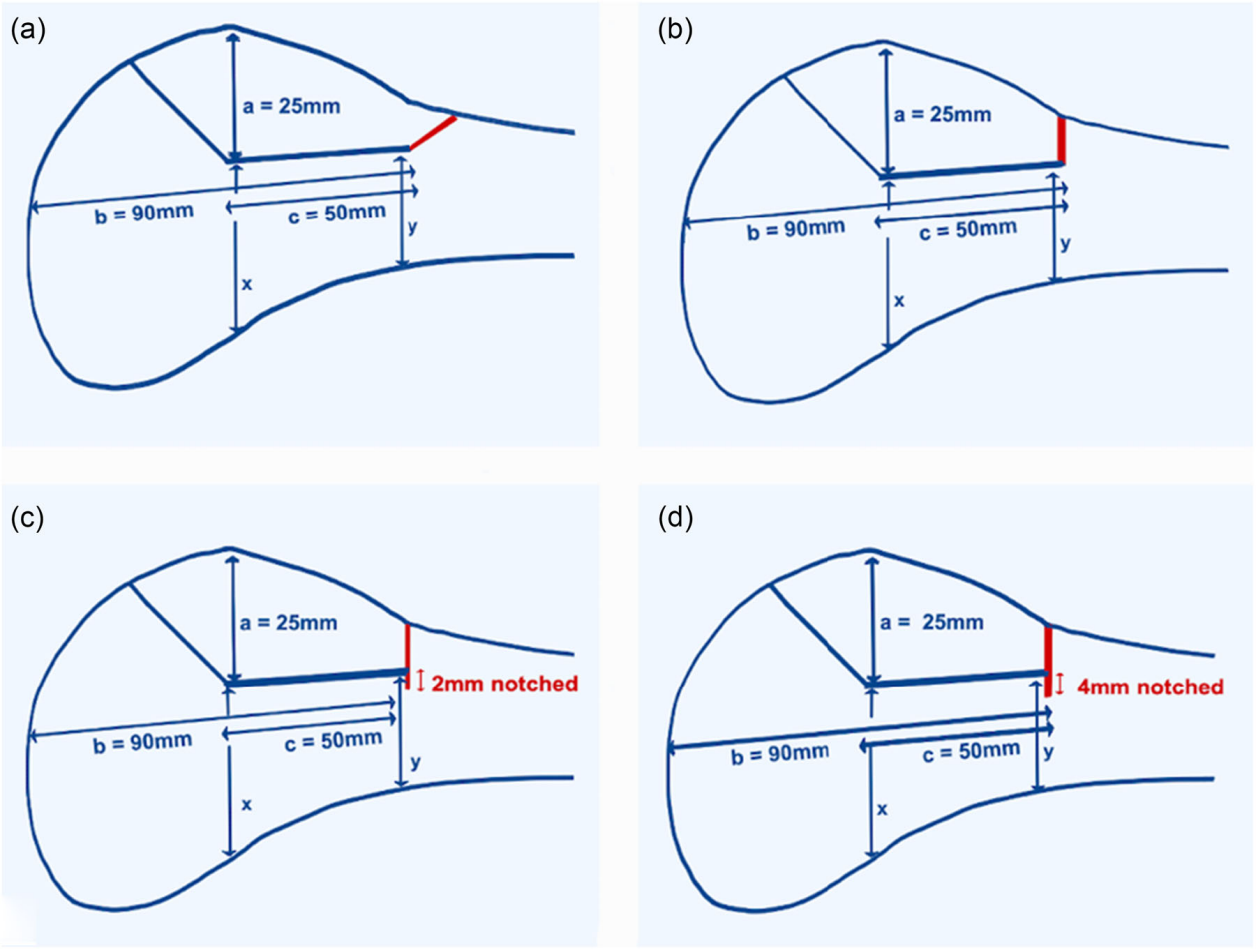
Schematic illustration of the four sawing technique groups for tibial tubercle osteotomy (TTO) (porcine proximal tibiae, sagittal view). (a) Oblique, (b) orthogonal without notching, (c) orthogonal 2 mm notch, (d) orthogonal 4 mm notch. Measurements: 25 mm cut depth, 90 mm distance from the eminentia intercondylaris to distal sawing cut, 50 mm osteotomy length, *x* = remaining proximal thickness after osteotomy, *y* = remaining distal tibial thickness after osteotomy.

For the osteotomy, specimens were secured in a clamping device. Fixation was ensured while avoiding clamping forces that could compromise the distal tibia. Osteotomy geometry was standardised across all groups (Figure 2). Key dimensions for the osteotomy included a tubercle fragment thickness of 25 mm (a) measured from the most anterior point, a distance of 90 mm between the intercondylar eminence and the distal osteotomy cut (b) and an osteotomy length of 50 mm in the coronal plane (c). Anatomical landmarks and osteotomy lines were marked before sawing. All osteotomies were performed using an oscillating saw (Battery Oscillator II, DePuy Synthes) with a standardised saw blade. According to the experimental protocol, either orthogonal or oblique distal cuts were performed. Oblique cuts were performed at an angle of 45° to the longitudinal axis of the tibia. Cortical notching was simulated in selected groups by creating a standardised, instrument‐controlled 2‐ or 4‐mm notch at the distal end of the orthogonal cut to mimic cortical weakening. The notch depths were validated using a manual vernier calliper by measuring the distance from the original cortical surface to the deepest point of the notch. After the osteotomy, all parameters were measured and documented. Residual tibial thickness was measured at the proximal and distal (*x*, *y*) aspects of the osteotomy to verify comparable cortical bone dimensions between the groups and to exclude residual thickness as a confounding factor for load‐to‐failure.

### Osteosynthesis techniques

**Figure 3:**
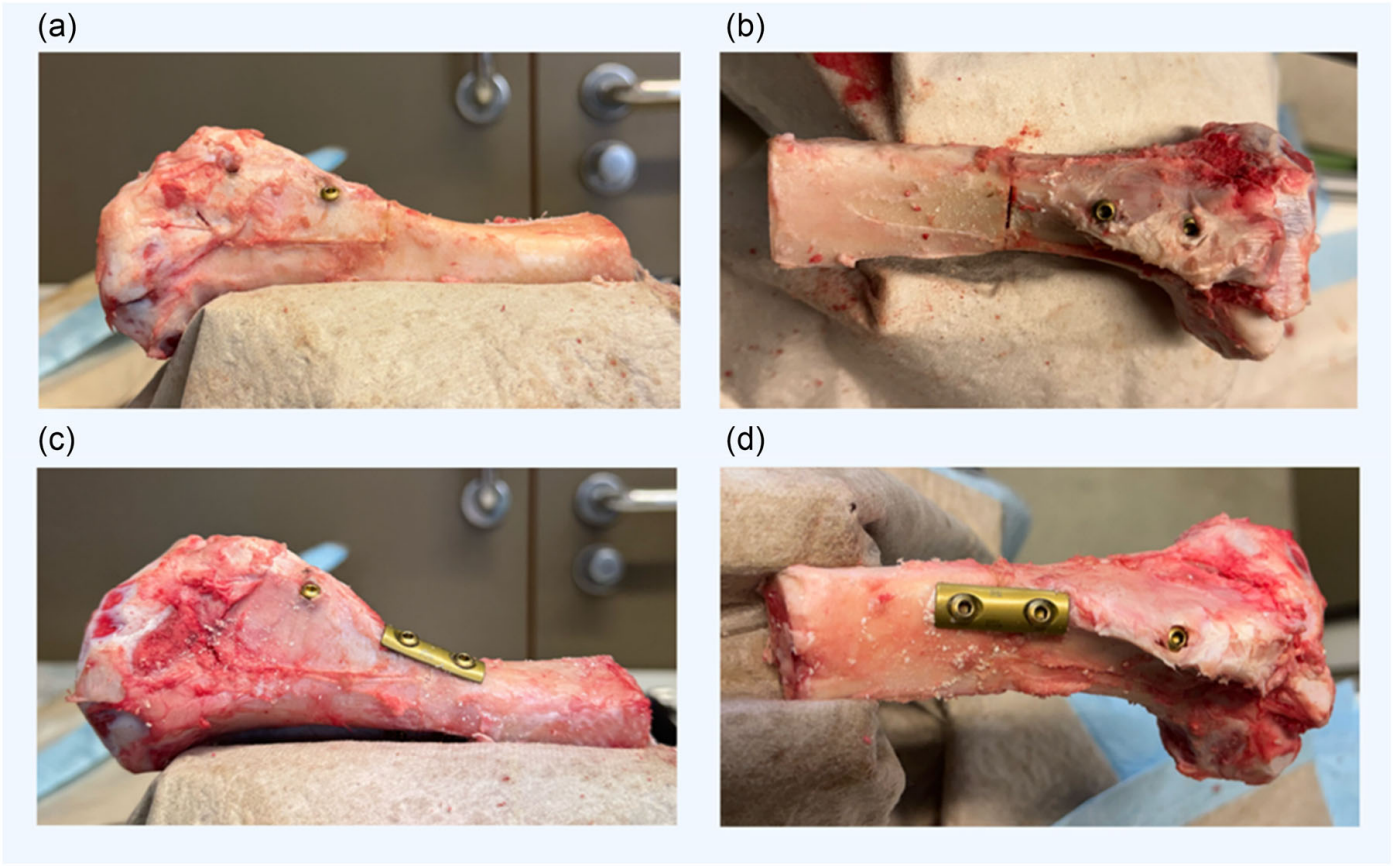
Osteosynthesis techniques after tibial tubercle osteotomy (TTO) with a 2‐mm notch. (a) Lateral view after osteotomy with refixation of the tibial tubercle using screw fixation with two bicortical screws. (b) Corresponding anterior view of the specimen shown in (a). (c) Lateral view after plate fixation using a two‐hole one‐third tubular plate and one bicortical screw. (d) Corresponding anterior view of the specimen shown in (c).

To evaluate the influence of different osteosynthesis techniques on tibial stability, two fixation techniques were compared. After the osteotomy, the tibial tubercle was reduced after distalization. Screw fixation was performed using two conventional bicortical lag screws (3.5 mm diameter), in accordance with the AO principles. Supplemental plate fixation was performed using a two‐hole one‐third tubular plate, which was used to bridge and compress the distal aspect of the osteotomy (Figure 3). Screw placement followed a standardised sequence, starting with the initial fixation of the plate proximally, then the insertion of the distal screw, followed by the proximal screw.

### Biomechanical testing setup

**Figure 4:**
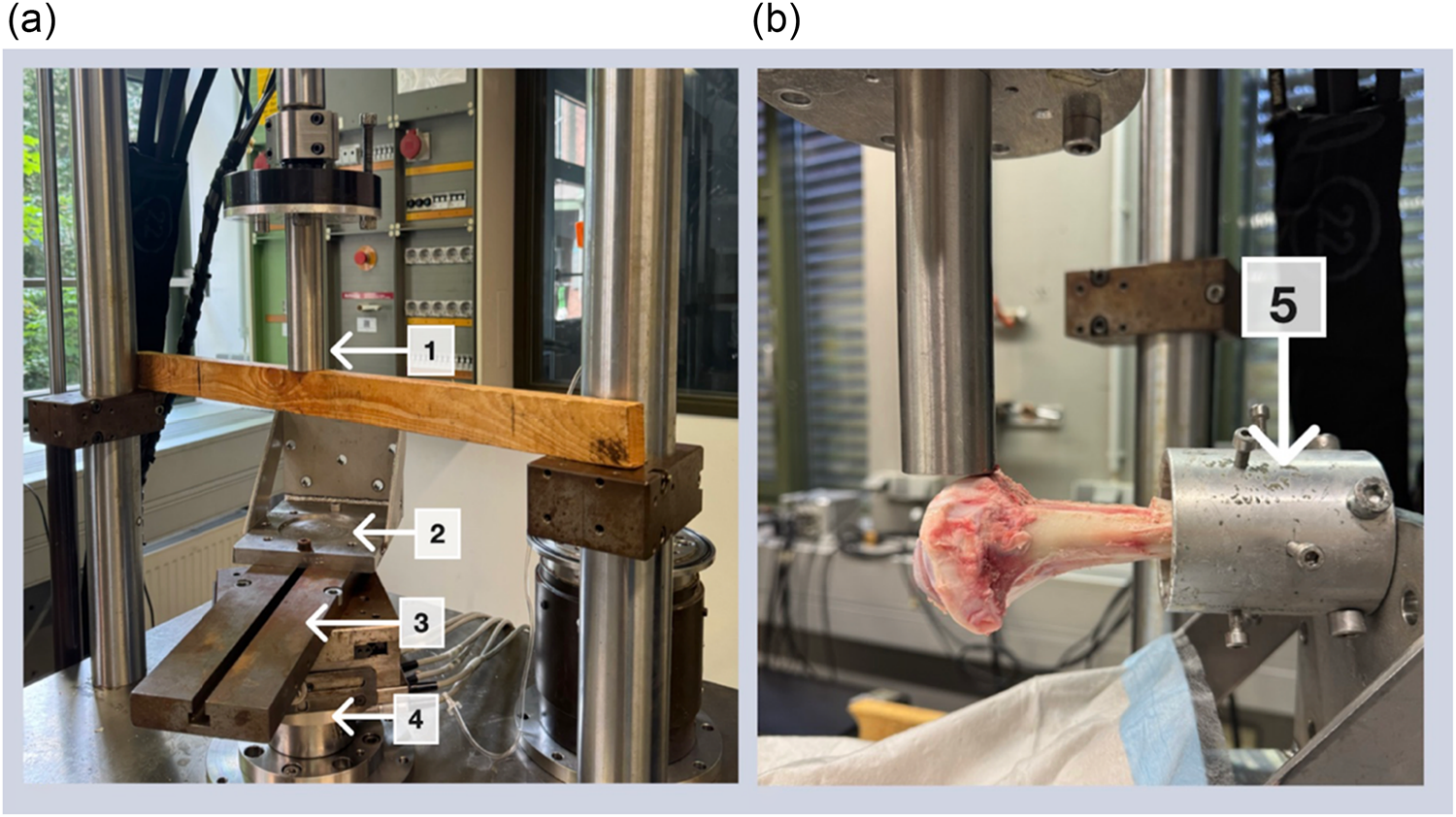
Servohydraulic testing machine (MTS Bionix 358). (a) Experimental setup of the MTS testing system. (b, c) Tibiae were fixed in a casting pot and mounted on the XY table. The loading indenter was positioned directly above the prepared tubercle surface and connected to the testing machine via the load cell. 1: Loading indenter; 2: 90° angle adaptor; 3: length‐adjustable base plate; 4: XY table for specimen fixation; 5: metal pot with embedded tibia.

After osteotomy, all specimens were embedded distally in a metal pot using polymethyl methacrylate (PMMA; Technovit 4004, Kulzer, Germany). Embedding was standardised to 20 mm beyond the distal osteotomy cut to ensure consistent conditions. The experimental setup was designed to approximate a simplified cantilever bending model. The distance between the point of load application and the embedding (lever arm, *l*) was kept constant across all specimens through the standardised embedding procedure. This ensured uniform loading conditions and allowed direct comparison of failure loads. Biomechanical testing was performed using a servohydraulic testing machine (MTS Bionix 358, MTS Systems, Eden Prairie). Axial load was applied with a flat indenter (35 mm diameter) aligned perpendicular to the tibial shaft. The embedded specimens were secured to an adjustable XY‐table to ensure precise vertical alignment (Figure 4). Load and displacement data were continuously recorded using the MTS FlexTest GT software.

## EXPERIMENTAL PROTOCOL

**Figure 5:**
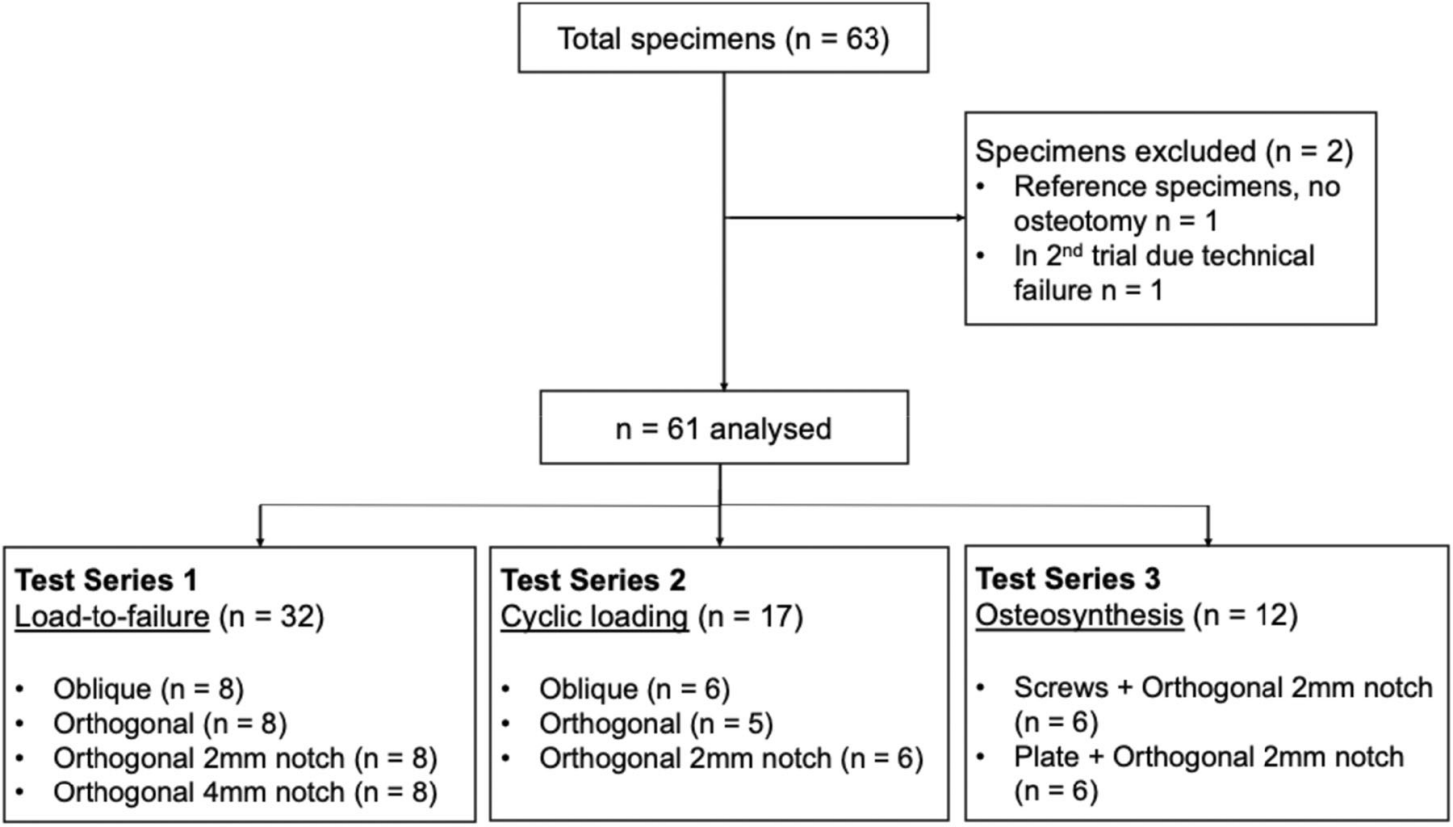
Flowchart illustrating the study design and specimen allocation across three independent biomechanical test series.

The specimens were allocated to three independent biomechanical test series. The study design and specimen distribution are illustrated in Figure 5.

### Test series 1—Load‐to‐failure analysis

**Figure 6:**
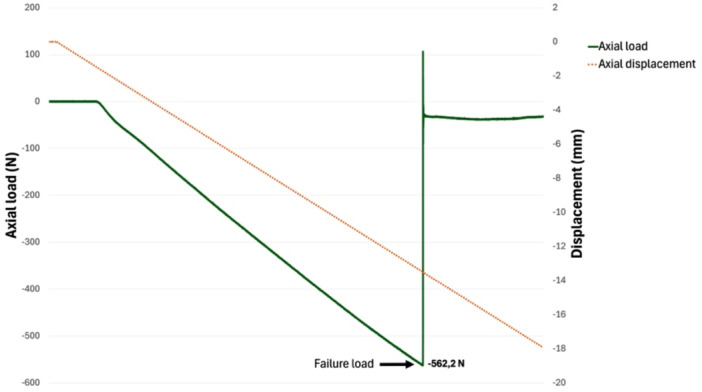
Representative load–displacement curve obtained during static load‐to‐failure testing. Failure load was defined as the first distinct drop in the load–displacement curve, indicating structural failure of the tibia. Compression is displayed as negative load values.

Four groups of distal cut orientations (*n* = 8 each) were tested and compared: oblique cut (Obl), orthogonal cut (Orth) without cortical notching, orthogonal cut with 2‐mm cortical notch (Orth2) and orthogonal cut with 4‐mm cortical notch (Orth4). One intact tibia served as a reference specimen. Specimens were tested axially in a displacement‐controlled two‐point bending configuration under axial loading at a rate of 0.5 mm/s to ensure controlled and reproducible testing conditions. Failure was defined as the first distinct drop in the load‐displacement curve, with compressive loads assigned negative values and tensile loads positive values (Figure 6).

### Test series 2—Cyclic loading analysis

In a second experimental series, cyclic loading was applied to evaluate fatigue fracture behaviour under increasing loads and to simulate repetitive submaximal loading conditions during the early postoperative period. Based on the maximum failure loads, Obl, Orth and Orth2 were tested (initially *n* = 6 per group). One Orth specimen was excluded prior to testing due to technical failure, resulting in a final sample size of 17 specimens. The Orth4 group was not included in the second test series because no significant differences in failure load were observed between Orth2 and Orth4 in test series 1. A constant preload of 50 N was applied to maintain continuous contact within the construct and to prevent complete unloading between cycles. Cyclic loading was performed at 1 Hz to approximate repetitive physiological loading conditions. The upper load limit started at 180 N as a submaximal load below the failure loads observed in test series 1, and increased continuously by 0.05 N per cycle until failure or a maximum load of 1000 N was reached. Failure during cyclic loading was defined as structural fracture of the tibia associated with a distinct drop in the load‐displacement curve and loss of construct integrity, resulting in termination of the test. The progressive increase in load was chosen to gradually challenge construct stability and assess fatigue resistance under increasing repetitive loading conditions. Specimens were continuously hydrated during those tests to prevent dehydration‐induced reduction of tibial bone stability.

### Test series 3—Osteosynthesis stability analysis

In a third experimental series, the influence of different osteosynthesis techniques on tibial bone stability was assessed. Specimens with a 2‐mm cortical notch were selected to ensure comparable baseline conditions, as the preceding tests demonstrated a significant reduction in stability in this group. Twelve porcine tibiae were analysed and allocated to two groups (lag screw fixation vs. plate fixation, *n* = 6 per group), as described above. All specimens were tested under static axial load‐to‐failure conditions using the same biomechanical protocol as applied in the first load‐to‐failure experiment. Implant‐related failure modes were documented.

## OUTCOME PARAMETERS

Load‐to‐failure was measured in N and defined as the value before structural failure. In cyclic testing, the number of cycles to failure was recorded. Axial displacement at failure (mm) was used to characterise deformation behaviour. Structural stiffness (N/mm) was calculated by linear regression of the linear elastic region of the load‐displacement curve between 20% and 60% of the maximum load. For specimens with multiphasic failure patterns, only the initial peak load was considered.

### Statistical analysis

Statistical analysis was performed using SPSS Statistics (IBM Corp.). The data were visually inspected for plausibility and outliers before analysis. Descriptive statistics were reported as mean and standard deviation (±SD). Normality of data distribution was assessed using the Shapiro–Wilk test. Group comparisons involving more than two groups were performed using one‐way analysis of variance (ANOVA) for normally distributed data or Welch's ANOVA in the presence of variance heterogeneity. For comparisons between two groups, independent samples *t*‐tests or Mann–Whitney *U* tests were applied, if appropriate. Effect sizes were calculated as partial eta squared (*η* ^2^). Correlation analyses between biomechanical parameters were performed using Pearson or Spearman correlation coefficients, depending on data distribution. The level of significance was set at *p* < 0.05. An a priori power analysis based on previously published biomechanical data indicated that eight specimens per group would provide 90% power to detect a 25% difference in load‐to‐failure with a significance level of *α* = 0.05 [6, 33]. For the secondary analyses, 17 specimens in the cyclic loading test series (*n* = 6/5/6) provided 99.6% power to detect the expected overall difference in cycles to failure at *α* = 0.05, whereas the osteosynthesis comparison (*n* = 6/6) was considered exploratory and powered only for large differences in failure load.

## RESULTS

**Figure 7:**
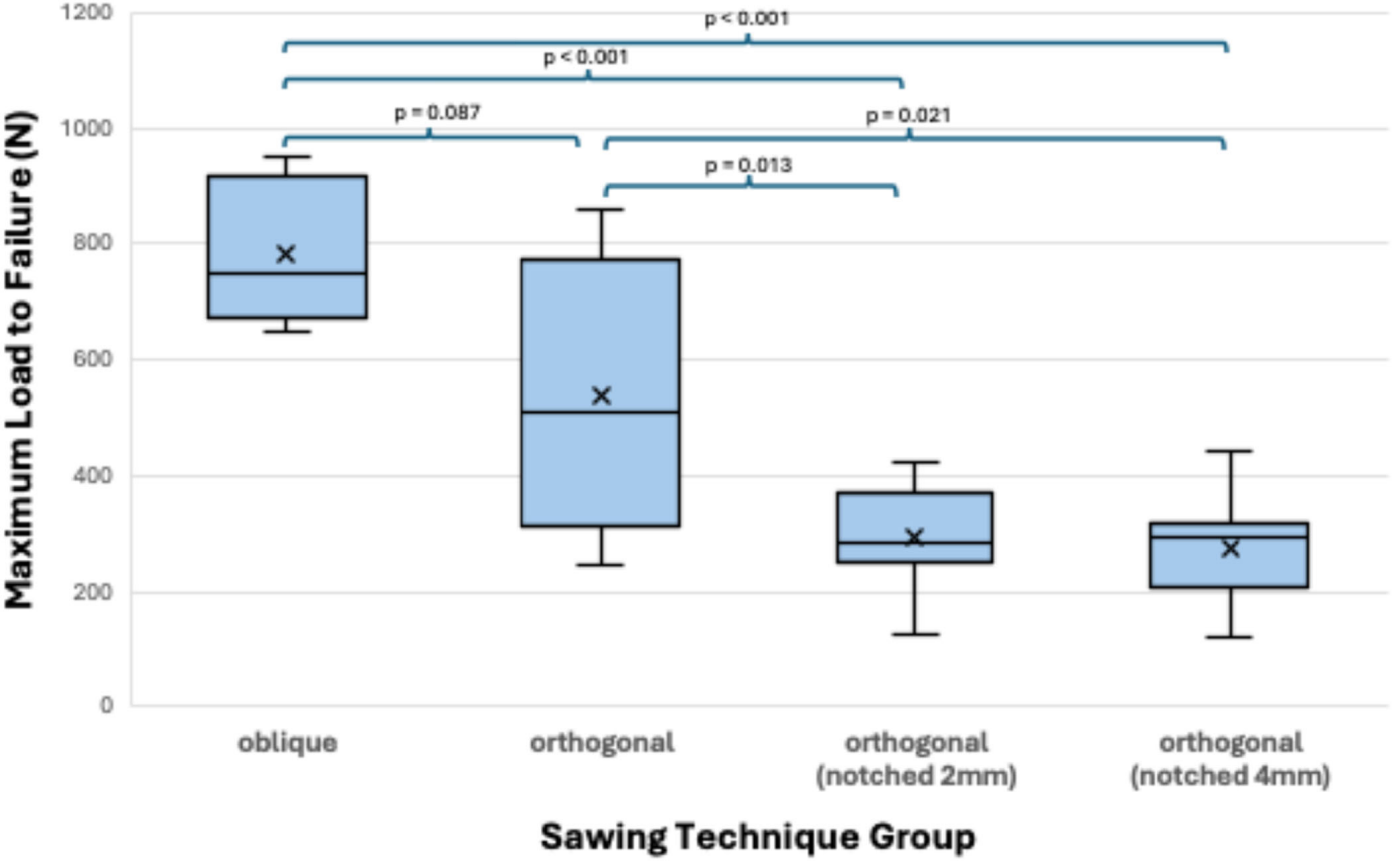
Boxplots showing the maximum load to failure for the different distal osteotomy cut configurations (first experimental series; *n* = 32).

In the load‐to‐failure analysis, 32 porcine tibiae were included. Seventeen specimens were analysed for fatigue fracture behaviour under cyclic loading, and 12 specimens were analysed for the comparison of osteosynthesis techniques. Normal distribution was confirmed for all parameters. In total, two specimens were not analysed. One intact specimen served as a reference, and one specimen was excluded prior to biomechanical testing because instability of the PMMA embedding resulted in premature specimen damage. The distribution of load‐to‐failure is illustrated in the boxplots (Figure 7). Outcome parameters are presented in Table 1.

**Table 1:** Biomechanical outcomes of different distal osteotomy cut orientations and fixation techniques.

Parameter	Oblique	Orthogonal	Orthogonal + 2‐mm notch		Orthogonal + 4‐mm notch	*p*‐value
Experiment 1—Static load‐to‐failure						
*n*	8	8	8		8	
Load‐to‐failure (*N*)	784.5 ± 118.3	538.8 ± 229.5	294.0 ± 91.8		274.6 ± 96.5	<0.001
Axial displacement (mm)	15.5 ± 2.0	10.8 ± 2.9	6.3 ± 1.4		6.6 ± 2.0	<0.001
Stiffness (*N*/mm)	56.9 ± 10.4	57.0 ± 18.8	47.1 ± 12.8		52.0 ± 19.2	0.549
Proximal residual thickness (mm)	25.5 ± 2.5	26.1 ± 2.4	25.8 ± 3.9		26.4 ± 3.9	0.952
Distal residual thickness (mm)	18.6 ± 1.5	16.5 ± 2.2	18.3 ± 2.1		17.1 ± 1.7	0.115
Experiment 2—Cyclic loading						
*n*	6	5	6			
Cycles to failure	5894 ± 2187	3142 ± 808	1400 ± 530			<0.001
Load‐to‐failure (*N*)	473.8 ± 106.3	332.0 ± 39.9	243.2 ± 22.3			<0.001
Experiment 3—Fixation techniques (2‐mm notch)						
			**Plate fixation**	**Screw fixation**		
*n*			6	6		
Load‐to‐failure (*N*)			373.6 ± 63.7	391.5 ± 154.7		0.802
Axial displacement (mm)			6.84 ± 1.10	7.08 ± 1.89		0.794
Stiffness (*N*/mm)			58.9 ± 18.4	58.1 ± 31.9		0.958
Proximal residual thickness (mm)			24.7 ± 2.4	24.2 ± 3.3		0.771
Distal residual thickness (mm)			16.2 ± 1.2	16.5 ± 1.9		0.752

*Note*: Values are presented as mean ± standard deviation. *p*‐Values refer to overall group comparisons within each experimental setup. *p*‐Values indicate overall group differences within each experimental setup (Welch‐ANOVA or Kruskal–Wallis test, as appropriate). Pairwise post hoc comparisons are reported in Section 5.

Load‐to‐failure testing revealed significant differences between distal osteotomy cut orientations (*p* < 0.001). Obl demonstrated higher failure loads than Orth, without reaching statistical significance (mean difference 245.7 N; 95% confidence interval [CI] −31.3 to 522.7 N; *p* = 0.087). Compared to Obl, cortical notching resulted in a significant reduction in load‐to‐failure (Obl vs. Orth2: mean difference 490.5 N; 95% CI 335.3–645.6 N; *p* < 0.001; Obl vs. Orth4: mean difference 509.9 N; 95% CI 353.2–667.7 N; *p* < 0.001). Similarly, Orth demonstrated significantly higher failure loads compared to Orth2 (mean difference 244.8 N; 95% CI 38.6–451.0 N; *p* = 0.013) and Orth4 (mean difference 264.2 N; 95% CI 58.0–470.4 N; *p* = 0.021). No significant difference was observed between Orth2 and Orth4 (mean difference 19.5 N; 95% CI −117.5 to 156.4 N; *p* = 0.975).

Axial displacement at failure differed significantly between groups (ANOVA, *p* < 0.001). Obl demonstrated greater axial displacement than Orth (mean difference 4.74 mm; 95% CI 1.64–7.85 mm; *p* = 0.001), Orth2 (mean difference 9.22 mm; 95% CI 6.12–12.32 mm; *p* < 0.001) and Orth4 (mean difference 8.88 mm; 95% CI 5.78–11.98 mm; *p* < 0.001). Orth also exhibited greater displacement than Orth2 (mean difference 4.47 mm; 95% CI 1.37–7.58 mm; *p* = 0.002) and Orth4 (mean difference 4.14 mm; 95% CI 1.03–7.24 mm; *p* = 0.004). No significant difference was detected between Orth2 and Orth4 (mean difference 0.34 mm; 95% CI −2.77 to 3.44 mm; *p* = 1.000). The axial displacement showed a strong positive correlation with load‐to‐failure (*r* = 0.829, *p* < 0.001).

One‐way ANOVA revealed no significant differences in structural stiffness between groups (*p* = 0.549; *η* ^2^ = 0.072), indicating similar baseline bone quality. Pairwise post hoc comparisons with Bonferroni correction showed no significant differences between any group combinations (all adjusted *p* = 1.000), with mean differences ranging from −9.9 to 9.9 N/mm and all 95% CIs crossing zero.

**Figure 8:**
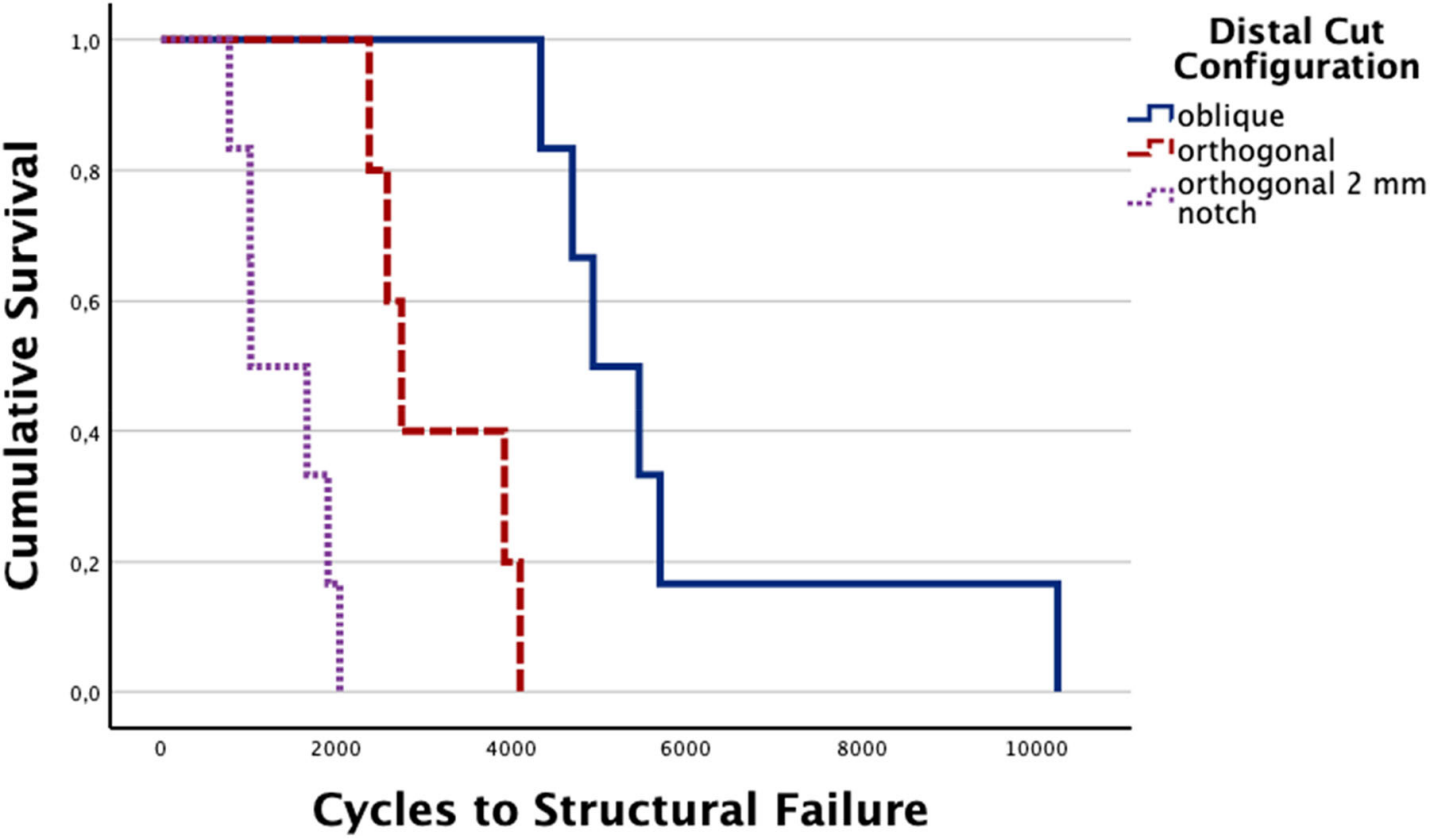
Kaplan–Meier curve depicting the number of cycles to structural failure.

During cyclic testing, Obl withstood significantly more cycles until failure compared to Orth (mean difference 2752 cycles; *p* = 0.004; Figure 8) and Orth2 (mean difference 4494 cycles; *p* = 0.002). Orth also withstood significantly more cycles than Orth2 (mean difference 1742 cycles; *p* = 0.004). Effect sizes were large for all pairwise comparisons (*r* = 0.93). No specimen reached the predefined maximum load threshold during cyclic testing.

Specimens with an oblique distal cut demonstrated the highest load to failure, whereas orthogonal cuts with cortical notching showed substantially reduced mechanical strength.

Obl demonstrated the longest survival time (mean 5894 ± 2187 cycles), followed by the Orth (3142 ± 808 cycles). Orth2 failed significantly earlier (1400 ± 530 cycles; *p* < 0.001). Orth4 was not included in the cyclic loading analysis due to the lack of significant differences in load‐to‐failure between Orth2 and Orth4 in test series 1.

Regarding the type of osteosynthesis, no significant differences were found between additional plating and lag screw fixation for any biomechanical outcome parameter. Load‐to‐failure was comparable between both techniques (mean difference 17.8 N; 95% CI −181.1 to 145.4 N; *p* = 0.802). However, compared with Orth2 without osteosynthesis, neither plate fixation (*p* = 0.060) nor screw fixation (*p* = 0.264) resulted in a significant increase in failure load. Similar observations were made for axial displacement at failure (*p* = 0.794) and construct stiffness (*p* = 0.958), with no differences between the two groups.

Residual tibial thickness at the proximal and distal aspects of the osteotomy was comparable in all groups. Proximal residual thickness ranged from 25.5 ± 2.5 mm in Obl to 26.4 ± 3.9 mm in Orth4, with no significant intergroup differences (ANOVA, *p* = 0.952; Levene's test *p* = 0.506). Similarly, distal residual tibial thickness was comparable across groups, with mean values ranging from 16.5 ± 2.2 mm in Orth to 18.6 ± 1.5 mm in Obl (ANOVA, *p* = 0.115; Levene's test *p* = 0.643), indicating comparable residual bone geometry across all groups.

Linear regression analysis demonstrated a significant positive association between distal residual tibial thickness and load‐to‐failure across all distal cut orientations (all *p* < 0.05). The increase in failure load per millimetre of residual thickness was highest in Orth (*β* = 90.4 N/mm; 95% CI 38.9–142.0 N/mm; intercept = −953.6 N; *R* ^2^ = 0.755; *p* = 0.005), followed by Obl (*β* = 69.2 N/mm; 95% CI 31.9–106.4 N/mm; intercept = −503.7 N; *R* ^2^ = 0.775; *p* = 0.004), Orth4 (*β* = 48.8 N/mm; 95% CI 21.6–76.0 N/mm; intercept = −561.0 N; *R* ^2^ = 0.762; *p* = 0.005) and Orth2 (*β* = 33.1 N/mm; 95% CI 3.1–63.1 N/mm; intercept = −310.4 N; *R* ^2^ = 0.548; *p* = 0.036), indicating that thicker residual bone was associated with greater stability.

Across all test series, fractures consistently extended from the distal osteotomy site in a distal and posterior direction, independent of fixation technique. No plate deformation or implant failure was observed. No primary implant‐related failure or plate deformation was observed.

## DISCUSSION

The most prominent finding was that the distal osteotomy cut orientation had a substantial impact on load‐to‐failure and fatigue failure in dTTO. Even minor cortical notching resulted in a relevant compromise of tibial stability. In addition, exploratory regression analyses demonstrated a positive association between specimen‐specific distal residual tibial shaft thickness and load‐to‐failure within the individual osteotomy groups. The choice of osteosynthesis technique did not affect tibial shaft stability under mechanical loading in cases of reduced stability due to cortical notching. Across all experimental setups, the oblique distal cut demonstrated the highest biomechanical stability, followed by the orthogonal cut, and notably reduced stability in the presence of cortical notching. The difference between oblique and orthogonal distal cuts did not reach statistical significance in the static load‐to‐failure analysis.

Perioperative tibial shaft fracture represents a rare but serious complication following TTO [1, 10, 31, 33]. In this regard, distal osteotomy orientation has been subject to previous investigations, has been associated with an increased fracture risk. Koeter et al. reported on a series of TTO for the treatment of patellar maltracking and recommended avoiding orthogonal distal cuts, as these may act as stress risers [19]. In a series of 101 TTOs, Luhmann et al. observed a comparably high rate of tibial shaft fractures (5.9%), but found lower fracture rates when using oblique distal cuts compared with orthogonal cutting techniques [21]. In another case series of 263 cases, Rood et al. observed two tibial fractures, using a V‐shaped osteotomy technique including an orthogonal distal cut [28]. Although the reported fracture rate was <1%, the authors suspected an association between orthogonal cutting and an increased fracture risk, which prompted the authors to reduce postoperative weight‐bearing in the applied rehabilitation protocol [28]. These observations are partially consistent with the findings of this study. With regard to the maximum load‐to‐failure, no significant differences were observed between orthogonal and oblique distal cut orientations. At the same time, orthogonal cuts withstood significantly fewer cycles compared to oblique cuts, until a tibial fracture occurred. Although this observation is inherently experimental, failure under cyclic loading may better reflect the repetitive loading conditions encountered during postoperative rehabilitation than a single load‐to‐failure event. However, the exact biomechanical mechanism underlying postoperative tibial fractures remains unclear. In recent literature, most of the existing data are based on retrospective clinical observations. Therefore, due to the lack of biomechanical studies on this subject, the comparability to other studies is limited. In particular, retrospective case series do not allow for causal analysis or adequate adjustment for potential confounders, including details of surgical technique when performing TTO.

In this study, technical aspects of dTTO were primarily hypothesised to influence the perioperative fracture risk. In this context, and based on the presented results, cortical notching at the distal aspect of the osteotomy was identified as a major risk factor for reduced tibial stability following (orthogonal) TTO. From a biomechanical perspective, such geometric discontinuities can act as stress concentrators in the anterior tibial cortex [5]. Accordingly, even small cortical notches (≥2 mm) resulted in a significant decrease in load‐to‐failure and reduced durability under cyclic loading. Notably, increasing notch depth from 2 to 4 mm did not result in further loss of stability. This may indicate that even limited disruption of the anterior tibial cortex is sufficient to create a relevant stress concentration, whereas further enlargement of the notch does not necessarily result in an additional measurable loss of construct stability under the present experimental conditions. However, given the limited sample size, a type II error cannot be excluded, and this finding should therefore be interpreted with caution. While this may appear to be a specific issue, cutting inaccuracies with oscillating saws represent a clinically relevant problem [3]. In a cadaveric study on cutting accuracy in total knee arthroplasties (TKA), Herregodts et al. observed saw blade excursions of 2.8–6.1 mm depending on the analysed aspect of the tibial plateau, even in experienced hands [16]. Although no such studies exist for osteotomies, these findings are likely applicable given the use of similar instruments [3]. Small cortical notches can easily go unnoticed intraoperatively, potentially making them particularly hazardous. Nevertheless, orthogonal cuts are commonly used in distalizing TTO, as the straight cut ends facilitate a controlled and measurable distalization [26]. In contrast, an oblique distal cut in dTTO may be more technically demanding, requiring either resection of a parallel fragment or trimming of the distal end with a rongeur.

Besides cortical notching, exploratory regression analyses suggested that greater distal residual tibial shaft thickness was associated with higher load‐to‐failure, particularly at the distal aspect of the osteotomy, which seems to represent a biomechanically vulnerable zone. This has direct clinical implications regarding the thickness of the tibial tubercle fragment, which has been proposed to be at least 7–10 mm, in order to achieve sufficient fragment stability for fixation [19, 20, 21, 27]. Conversely, thin tubercle fragments may increase the risk of fracture following screw fixation [32]. The findings of this study indicate that fragment thickness should be selected in relation to the overall tibial geometry to avoid compromising the residual tibial shaft thickness. Furthermore, residual tibial shaft thickness may represent an additional independent risk factor, not directly related to cortical notching. In line with this, no differences in residual tibial shaft thickness were observed between the analysed groups. However, these findings are based on subgroup‐specific regression analyses with a limited number of specimens and should therefore be considered exploratory. Further biomechanical investigations are required to confirm the observed associations.

Concerning fixation stability, the choice of osteosynthesis represents another clinically relevant aspect. While previous studies reported improved stability of the osteotomy fragment with supplemental plate fixation, these investigations primarily addressed fragment displacement rather than tibial shaft integrity [14, 30]. As additive plate fixation provides additional bridging of the anterior cortex, a stabilising effect on tibial shaft stability may be assumed. In the presented study, no relevant differences in tibial shaft stability were observed between screw fixation and additional plate fixation under the applied loading conditions. More specifically, the observed fracture patterns were not different from the patterns in specimens without osteosynthesis, occurring at the distal aspect of the osteotomy. Within the limitations of the applied experimental setup, additional plating using a one‐third tubular plate did not demonstrate a measurable stabilising effect on tibial stability.

## LIMITATIONS

This study has some limitations. First, as an in vitro investigation, the experimental setup cannot fully replicate in vivo conditions. Dynamic muscle forces, ligamentous restraints, joint kinematics and biological healing processes were not simulated. Consequently, the findings primarily reflect early postoperative mechanical behaviour rather than mid‐ or long‐term clinical stability. Second, loading was limited to a simplified cantilever bending model under axial loading. Although bending moments play a relevant role in perioperative tibial fractures during gait, this setup represents only one component of the complex, multi‐directional loading conditions acting on the tibia in vivo. Clinical fracture mechanisms following dTTO are likely influenced by a combination of axial compression, quadriceps traction, torsional forces, knee flexion angle and dynamic gait‐related loading. Therefore, the results should be interpreted within the limitations of a controlled biomechanical experiment. Third, while porcine bone is an established surrogate in orthopaedic biomechanics with comparable cortical structure and mechanical properties, morphological differences exist, particularly regarding the tibial tubercle. To ensure interaction with the diaphyseal cortex, a greater osteotomy depth was required compared to typical human dTTO procedures. Therefore, absolute failure load values should be interpreted with caution, whereas relative differences between experimental groups are considered representative. Furthermore, studies with larger sample sizes could help to underline the findings of this study and potentially improve statistical power for comparison of osteosynthesis. Finally, dTTO was only simulated and not fully reproduced, limiting the assessment of potential technical challenges associated with clinical execution.

Within the limitations of an in vitro study, the present study provides quantitative biomechanical evidence supporting the relevance of distal cut orientation, cortical integrity and residual tibial shaft thickness for construct stability. These findings reinforce the importance of careful surgical technique and individualised consideration of tibial anatomy in dTTO.

## CONCLUSION

In this porcine model, cortical notching significantly reduced tibial stability and should be avoided during TTO. Oblique distal cuts showed greater resistance to cyclic loading, while no significant difference was observed in static load‐to‐failure. The osteosynthesis technique did not affect load‐to‐failure.

## AUTHOR CONTRIBUTIONS

Eiken Senkbeil contributed to study conception and design, data collection, data analysis and manuscript drafting. Kay Sellenschloh contributed to biomechanical testing and provided technical and methodological support. Sara Checa, Karl‐Heinz Frosch and Matthias Krause contributed to study design and critically revised the manuscript. Jannik Frings conceived and supervised the study and contributed to study design and manuscript revision. All authors read and approved the final manuscript.

## CONFLICT OF INTEREST STATEMENT

Karl‐Heinz Frosch and Matthias Krause consultancy agreement with Arthrex (Naples, FL, USA). Jannik Frings consultancy agreement with Conmed (Largo, FL, USA). The remaining authors declare no conflicts of interest.

## ETHICS STATEMENT

Ethical approval was not required, as animal by‐products from the food industry were used and would otherwise have been discarded.

## Data Availability

Research data are not shared.
